# A knowledge-based intensity-modulated radiation therapy treatment planning technique for locally advanced nasopharyngeal carcinoma radiotherapy

**DOI:** 10.1186/s13014-020-01626-z

**Published:** 2020-08-03

**Authors:** Penggang Bai, Xing Weng, Kerun Quan, Jihong Chen, Yitao Dai, Yuanji Xu, Fasheng Lin, Jing Zhong, Tianming Wu, Chuanben Chen

**Affiliations:** 1 Department of Radiation Oncology, Fujian Cancer Hospital & Fujian Medical University Cancer Hospital, Fuzhou, China; 2 Department of Radiation Oncology, Fujian Medical University Union Hospital, Fuzhou, China; 3 School of Nuclear Science and Technology, University of South China, Hengyang, China; 4 Department of Radiology, Fujian Cancer Hospital & Fujian Medical University Cancer Hospital, Fuzhou, China; 5 Department of Radiation and Cellular Oncology, The University of Chicago Medicine, Chicago, USA

**Keywords:** Knowledge-based, Intensity-modulated radiation therapy, Automated planning, Nasopharyngeal carcinoma

## Abstract

**Background:**

To investigate the feasibility of a knowledge-based automated intensity-modulated radiation therapy (IMRT) planning technique for locally advanced nasopharyngeal carcinoma (NPC) radiotherapy.

**Methods:**

One hundred forty NPC patients treated with definitive radiation therapy with the step-and-shoot IMRT techniques were retrospectively selected and separated into a knowledge library (*n* = 115) and a test library (*n* = 25). For each patient in the knowledge library, the overlap volume histogram (OVH), target volume histogram (TVH) and dose objectives were extracted from the manually generated plan. 5-fold cross validation was performed to divide the patients in the knowledge library into 5 groups before validating one group by using the other 4 groups to train each neural network (NN) machine learning models. For patients in the test library, their OVH and TVH were then used by the trained models to predict a corresponding set of mean dose objectives, which were subsequently used to generate automated plans (APs) in Pinnacle planning system via an in-house developed automated scripting system. All APs were obtained after a single step of optimization. Manual plans (MPs) for the test patients were generated by an experienced medical physicist strictly following the established clinical protocols. The qualities of the APs and MPs were evaluated by an attending radiation oncologist. The dosimetric parameters for planning target volume (PTV) coverage and the organs-at-risk (OAR) sparing were also quantitatively measured and compared using Mann-Whitney U test and Bonferroni correction.

**Results:**

APs and MPs had the same rating for more than 80% of the patients (19 out of 25) in the test group. Both AP and MP achieved PTV coverage criteria for no less than 80% of the patients. For each OAR, the number of APs achieving its criterion was similar to that in the MPs. The AP approach improved planning efficiency by greatly reducing the planning duration to about 17% of the MP (9.85 ± 1.13 min vs. 57.10 ± 6.35 min).

**Conclusion:**

A robust and effective knowledge-based IMRT treatment planning technique for locally advanced NPC is developed. Patient specific dose objectives can be predicted by trained NN models based on the individual’s OVH and clinical TVH goals. The automated planning scripts can use these dose objectives to efficiently generate APs with largely shortened planning time. These APs had comparable dosimetric qualities when compared to our clinic’s manual plans.

## Background

Radiation therapy treatment planning for nasopharyngeal carcinoma (NPC) is often challenged by the convoluted target volume and many adjacent organs at risk (OAR) [1]. Intensity-modulated radiation therapy (IMRT) technique has been considered as a common treatment for NPC, because it delivers highly conformal doses to the targets and effectively spares the OARs, potentially improving the local control rate and reducing radiation-related toxicities [2]. However, it is time-consuming to manually generate an IMRT plan due to its intrinsic trial-and-error process. In addition, IMRT plan quality may be inconsistent due to the inhomogeneous knowledge and experience level of the planners [3]. Hence, it is of great need to develop highly efficient automated planning techniques to consistently generate high quality plans.

In general, automated planning techniques are either algorithm based on some optimization methods [4–10] or knowledge based on prior plan data [11–19]. The knowledge-based techniques usually involve machine learning methods, which demonstrated their utility in improving treatment planning quality and efficiency. Some commercial modules can generalize a dose volume histogram (DVH) estimation model, from which treatment plans can be generated semi- or fully-automatically [11–13]. An in-house knowledge-based treatment planning technique has also been developed and proved effective in fully automating IMRT plans [20], using the overlap volume histogram (OVH) information [21]. One study recruited 138 head-and-neck patients but the inclusion of NPC patients was unknown [20]. Furthermore, all of these studies had not been exclusively applied to the treatment planning of locally advanced NPC patients [4–19]. The efficacy of the knowledge-based autoplan technique for locally advanced NPC treatment planning still needs further investigation due to the particular challenges from the tumor and OAR anatomy in this disease.

In our institution, we developed a knowledge-based IMRT treatment planning technique for locally advanced NPC based on a neural network (NN) machine learning model. The NN model correlated an individual patient’s OVH with the corresponding plan optimization dose objectives by learning from a cohort of similar locally advanced NPC patients. A set of Perl scripts were developed to bridge the NN model predicted patient specific dose objectives to the treatment planning system for plan optimization and dose calculations.

## Methods

### Patient libraries

Consecutive 140 locally advanced NPC patients treated with definitive IMRT at Fujian Cancer Hospital between July 2016 and September 2018 were retrospectively selected and chronologically separated into a knowledge library (*n* = 115) and a test library (*n* = 25). Only NPC patients with bilateral cervical lymph nodes metastases were included. All patients were diagnosed and staged by pretreatment enhanced magnetic resonance imaging (MRI) according to the Chinese 2008 staging system for NPC [22, 23]. Each patient was immobilised in a supine position with a thermoplastic mask and underwent contrast enhanced computed tomography (CT) (Brilliance CT Big Bore; Philips Medical Systems Inc., Cleveland, OH, USA) at a 3-mm slice spacing from the skull vertex to the level of 2 cm below the clavicles. Volume delineation was performed on the CT images in the Pinnacle3 treatment planning system (TPS) (Philips Radiation Oncology Systems, Madison, WI) after a CT-MRI fusion.

The target volumes were delineated using an institutional treatment protocol defined as following: the primary nasopharyngeal tumor (GTV_T) and definitive bilateral lymph nodes (GTV_NL and GTV_NR), as determined by clinical information, endoscopic examinations and radiography including CT and MRI. The clinical target volumes (CTVs) included high-risk regions (CTV1), low-risk regions (CTV2), and bilateral low-risk nodal regions (CTV_NL and CTV_NR). The CTV1 included GTV plus 5- to 10-mm margin. The CTV2 was designed for potentially involved regions and encompassed the entire CTV1. Each target volume was expanded by 3 mm to generate the planning target volume (PTV) in consideration of the setup error, geometric uncertainties and patient movement. In total, for each patient, seven target volumes (GTV_T_P, CTV1_P, CTV2_P, GTV_NL_P, GTV_NR_P, CTV_NL_P and CTV_NR_P) and ten OARs (left/right parotid, brainstem, spinal cord, left/right optic lens, left/right optic nerves, pituitary and optic chiasm) were delineated. A total dose of 69.96 Gy in 33 fractions at 2.12 Gy/fraction to the GTV_T_P, 66 Gy at 2 Gy/fraction to the GTV_NL_P/GTV_NR_P, 61.05 Gy at 1.85 Gy/fraction to the CTV1_P, 56.1 Gy at 1.7 Gy/fraction to the CTV2_P/CTV_NL_P/CTV_NR_P were prescribed.

### Manual planning

All patient treatment plans in the knowledge and test libraries were manually generated by a single experienced physicist. All plans were optimised in Pinnacle3 9.2 for treatment delivery by an Elekta Synergy accelerator using seven equally spaced coplanar 6MV photon beams (210°, 260°, 310°, 0°, 52°, 104°, and 156°). During treatment planning, auxiliary structures were generated to be used in objectives parameters (Table 1).

**Table 1:** Auxiliary structures for treatment planning

Structure	Generation approach
CTV1_GTV	avoiding GTV_T_P from CTV1_P
CTV2_CTV1	avoiding CTV1_P from CTV2_P
CTV_GTV_NL_P	avoiding GTV_NL_P from CTV_NL_P
CTV_GTV_NR_P	avoiding GTV_NR_P from CTV_NR_P
CTV_ALL	integrating CTV2_P, CTV_NL_P and CTV_NR_P
R5200	5-mm-wide rings coming from the 5 mm and 10 mm extension of the CTV_ALL
R4500	5-mm-wide rings coming from the 10 mm and 15 mm extension of the CTV_ALL
R3600	10-mm-wide ring coming from the 15 mm - 25 mm extension of the CTV_ALL
R3100	between body contour and 25 mm extension of the CTV_ALL

Direct machine parameter optimization (DMPO) was set for all beams with 9 cm^2^ minimum segment area, 9 minimum segment monitor unit (MU) and up to 60 maximum segments. The manual plans (MPs) in both libraries followed the institutional locally advanced NPC planning criteria shown in Table 2. To achieve these criteria, objectives shown in Table 3 were used as a starting point for planning. The type, volume and weight for regions of interest (ROIs) were preset and not allowed to change. Only the target dose objectives are tunable to improve plan quality. All the MPs required the planner’s best effort to lower the OAR doses by only adjusting the target dose values while maintaining the PTVs’ dose coverage. This iterative process shall be repeatedly executed until no further improvement can be made.

**Table 2:** The criteria of regions of interest for manual IMRT planning

Regions of interest	Criteria
GTV_T_P	68.96Gy < D_95_ < 70.96Gy
CTV1_P	D_95_ > 61.05Gy
CTV2_P	D_95_ > 56.1Gy
GTV_NL_P/GTV_NR_P	65.5Gy < D_95_ < 67Gy
CTV_NL_P/CTV_NR_P	D_95_ > 52.8Gy
left/right parotid	V_30_ < 50%
brainstem	D_1cc_ < 65Gy
spinal cord	D_1_ < 45Gy
left/right optic lens	Dmax<8Gy
left/right optic nerves	Dmax<62Gy
pituitary	Dmax<66Gy
optic chiasm	Dmax<66Gy

*Dx* Received dose corresponding to x% of volume, *Vx* Percentage volume corresponding to x Gy, *Dxcc* Received dose corresponding to x cubic centimeters

**Table 3:** Objective parameters

Region of interest	Objective parameters			
	type	Target dose	volume	weight
GTV_T_P	MinDVH	(1)	98	90
	MaxDVH	=MinDVH of GTV_T_P + 100	2	80
	UniformDose	= MinDVH of GTV_T_P + 50	/	75
CTV1_GTV	MinDVH	(2)	98	85
	MaxDVH	=MinDVH of GTV_T_P	2	80
CTV2_CTV1	MinDVH	(3)	98	85
	MaxDVH	6100	2	80
GTV_NL_P/GTV_NR_P	MinDVH	(4)(5)	98	90
	MaxDVH	=MinDVH of GTV_NL_P/ GTV_NR_P + 100	2	80
	UniformDose	=MinDVH of GTV_NL_P/ GTV_NR_P + 50	/	75
CTV_GTV_NL_P/CTV_GTV_NR_P	MinDVH	(6)(7)	98	85
	MaxDVH	6600	2	80
left/right parotid	MaxDVH	(8)(9)	50	50
brainstem	MaxDVH	(10)	0	50
spinal cord	MaxDVH	(11)	0	50
left/right optic lens	MaxDVH	(12)(13)	0	50
left/right optic nerves	MaxDVH	(14)(15)	0	50
pituitary	MaxDVH	(16)	0	50
optic chiasm	MaxDVH	(17)	0	50
R5200	MaxDVH	(18)	2	50
R4500	MaxDVH	(19)	2	50
R3600	MaxDVH	(20)	2	50
R3100	MaxDVH	(21)	2	50

(): 21 target dose objectives marked in the parentheses

(/ : None)

### Neural network model

The patients in the knowledge library were equally and chronologically divided into 5 groups, each group with 23 patients. A 5-fold cross validation scheme was adopted to generate 5 NN machine learning models. Each model was used to validate one group by training the other 4 groups. The output dose objectives for patients in the test library were obtained by taking the mean of the 5 dose objectives generated from the 5 models.

**Fig. 1:**
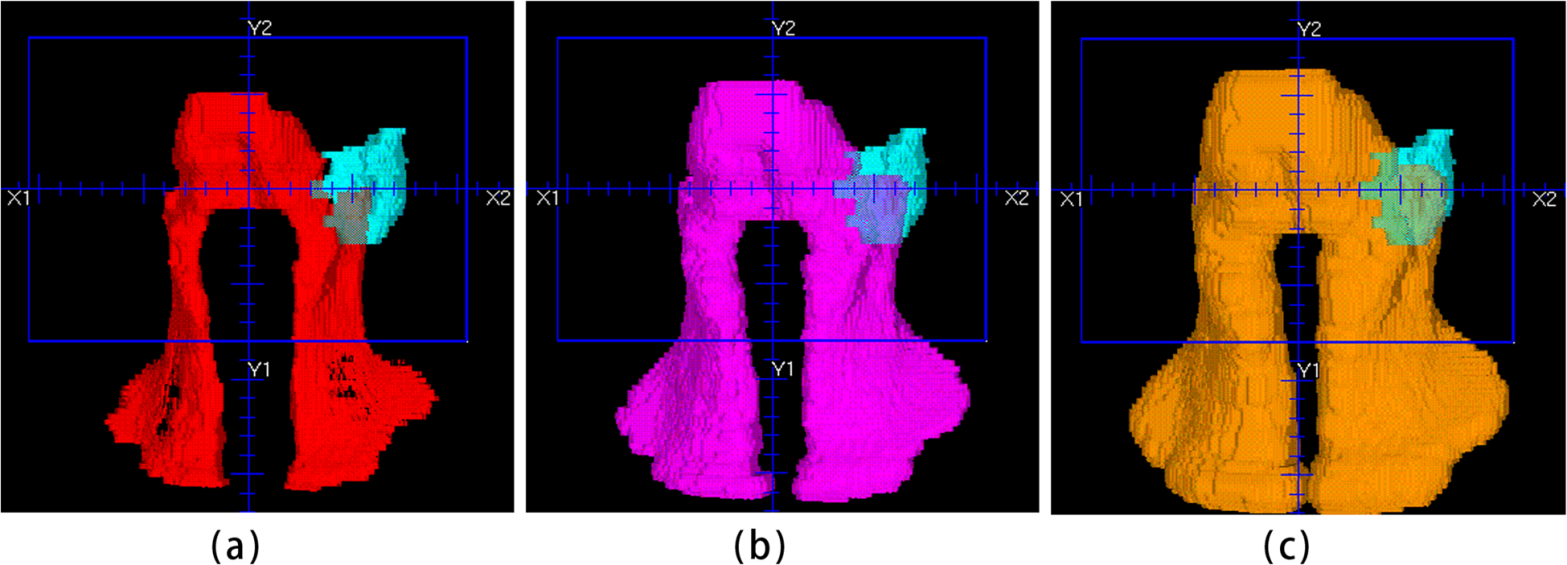
The overlapping between the left parotid (sky blue) and: (**a**) the CTV-ALL contracted with a distance of 5 mm (red); (**b**) the initial CTV-ALL (purple); (**c**) the CTV-ALL expanded with a distance of 5 mm (tan). The overlap volume fraction is defined as the overlapping volume divided by the volume of the left parotid

The details of how to build our NN model were given in this paragraph. For all patients in the knowledge library, their OVH, target volume histogram (TVH) and dose objective values were extracted and normalised. The OVH essentially defines the overlapping volume fraction between an OAR and a uniformly contracted/expanded PTV (see Fig. 1). It acts as a visualisable descriptor depicting the three-dimensional anatomical relationships between an OAR and the tumor volumes into the two-dimensional Cartesian coordinate system, which can be conveniently used as inputs to an NN model. The TVH indicates the uniformly contracted or expanded PTV. Each NPC patient in the knowledge library had 20 OVH, 5 TVH, and one set of 21 dose objectives. Both OVH and TVH had 11 values, starting from a zero or negative (contraction) distance to an ending positive distance (expansion) with a fixed step size (see Table 4). Our 3-layer NN model had consisted 275, 184 and 21 nodes in its input, hidden and output layer respectively, taking OVH and TVH values as inputs and returning dose objectives as desired outputs. The model learned by refining their node-to-node link weights between two neighboring layers to minimize the cost function defined as the mean squared error between the trained and known value on each output node.

**Table 4:** Overlap volume histogram (OVH) and target volume histogram (TVH) used as inputs to build neural network model

**OVH**				
OAR	Target volume	Starting distance (cm)	Step size (cm)	ending distance (cm)
Left/right parotid	CTV_ALL	− 1.0	0.2	1
Brainstem	CTV_ALL	−1.0	0.2	1
Spinal cord	CTV_ALL	0	0.2	2
Left/right optic lens	CTV_ALL	0	0.4	4
Left/right optic nerves	CTV_ALL	−1.0	0.3	2
Pituitary	CTV_ALL	−1.0	0.3	2
Optic chiasm	CTV_ALL	−1.0	0.3	2
CTV1_P	GTV_T_P	−1.0	0.2	1
CTV2_P	CTV1_P	−1.0	0.2	1
CTV_NL_P	GTV_NL_P	−1.0	0.2	1
CTV_NR_P	GTV_NR_P	−1.0	0.2	1
Left parotid	GTV_NL_P	−1.0	0.3	2
Right parotid	GTV_NR_P	−1.0	0.3	2
Left/right parotid	GTV_T_P	0	0.3	3
Pituitary	GTV_T_P	0	0.3	3
Optic chiasm	GTV_T_P	0	0.3	3
**TVH**				
Target volume		Starting distance (cm)	Step size (cm)	ending distance (cm)
GTV_T_P		−1.0	0.2	1
GTV_NL_P		−1.0	0.2	1
GTV_NR_P		−1.0	0.2	1
CTV1_P		−1.0	0.2	1
CTV2_P		−1.0	0.2	1

The NN modeling was run by Spyder (a python integrated development environment) on a personal computer with an Intel (i7-2630QM) CPU with 2 GHz main frequency. The model learning rate affects how big a step we update our model weights and values to move towards the minimum output error. The rate was set to 0.02 and model iteration time set to 2500. The choice of these parameters yielded satisfactory results in this feasibility study with relatively short training time. During test, the trained model simply calculated a set of patient specific dose objectives based on the OVH and TVH values.

### Automated planning

**Fig. 2:**
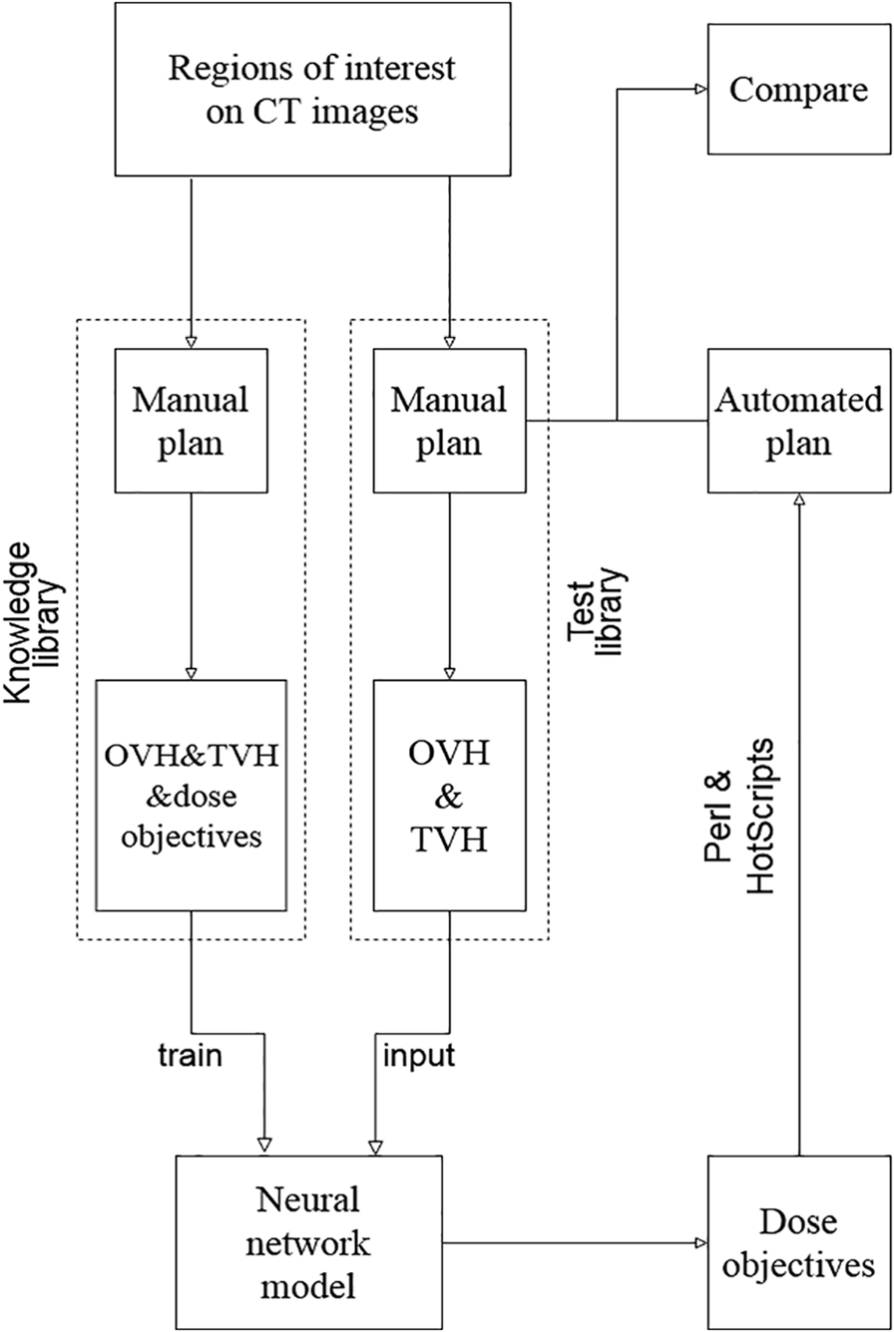
The flow chart of knowledge-based IMRT treatment planning technique for locally advanced nasopharyngeal carcinoma

Automated plans (APs) were all generated by an in-house developed Perl and HotScripts planning scripts in Pinnacle3 9.2. It automated the entire planning process including additional structure generation, beam and optimization parameters setup, and the final inverse optimization. This script also received planning parameters of gantry angle, beam energy, beam modality, treatment isocenter placing, prescription, number of fractions, isodose lines for visualization, IMRT optimization type, maximum number of segments, minimum segment area, minimum segment MU, max iteration (100) and convolution dose iteration at 40th. Finally, the script incorporated the derived dose objectives before the APs were automatically generated with a single loop of iteration of the planning process. An overview of our proposed process was presented in Fig. 2.

### Plan comparison and statistical analysis

The AP and the MP of each patient from the test library were all blindly reviewed and rated by one attending radiation oncologist in our institute by evaluating both DVH and dose distribution. Grade C indicated an inferior plan quality which is considered clinically unacceptable. A grade B plan was deemed just about acceptable and grade A suggested a superior plan where the DVH and dose distribution were more desirable. Similar quality plans could be deemed comparable and rated the same. The ratings for both the APs and MPs in the test library were compared by McNemar-Bowker tests using Statistical Package for the Social Sciences (SPSS 21.0; SPSS Inc., Chicago, IL, USA) software. The reviewer also recorded the numbers of ROIs achieving the given criteria to be compared between the APs and MPs.

SPSS 21.0 was also used for statistical analysis. The dose parameters in Table 2 were included in the statistical analysis. Mann-Whitney U test was performed to compare dose parameters of the APs and MPs. D_x_ was the received dose corresponding to x% of volume. D_5_ was used to evaluate the high dose in PTV. V_30_ was the percentage volume receiving 30 Gy dose. Conformity index (CI = (V_PTV region receiving prescription dose_/V_PTV_)* (V_PTV region receiving prescription dose_/V_Prescription dose_)) and homogeneity index (HI = D5/D95) were calculated for PTV evaluation. Furthermore, planning duration and MU per fraction were also analysed for both the APs and MPs. The alpha level was set at 0.05 and the Bonferroni correction was also applied to control type I error probability. Since 32 tests were carried out in this analysis, it was considered statistically significant when *P* < 0.0015.

## Results

### Plan quality comparison

**Fig. 3:**
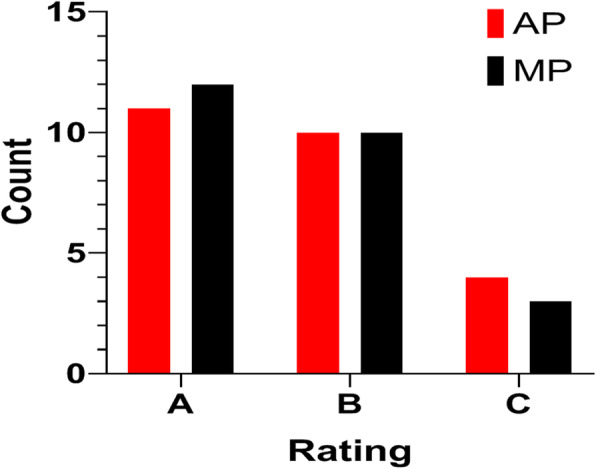
Blind review on plan quality between automated plans (APs) and manual plans (MPs) for the 25 NPC patients in the test library

In the blind test, 11 APs were rated A, 10 rated B, and 4 rated C, while 12 MPs were rated A, 10 rated B, and 3 rated C (see Fig. 3). The APs and MPs had the same rating in 19 out of 25 patients. APs were rated better for two patients and worse for four patients. The McNemar-Bowker test result showed that there existed no difference between the rating distribution of AP and MP with a *P* value of 0.549.

### ROI meeting criteria

The numbers of PTVs and OARs achieving the given criteria are listed in Table 5. For no less than 80% of the patients from the test library, the PTV coverage met the criteria in both the APs and MPs. Particularly, CTV_NL_P and CTV_NR_P of all the APs and MPs achieved their given criteria. GTV_T_P remained the most challenging PTV, since the number of GTV_T_P D_95_ achieved the given criteria was 20 and 22 in APs and MPs, respectively.

**Table 5:** The comparison between automated and manual IMRT plans for 25 patients with locally advanced nasopharyngeal carcinoma

ROI	Parameter	Criteria	Number		Parameter		
			AP	MP	AP (mean ± SD)	MP (mean ± SD)	*P*
GTV_T_P	D_5_(Gy)				76.27 ± 0.59	76.43 ± 0.98	0.756
	D_95_(Gy)	68.96Gy ~ 70.96Gy	20	22	69.61 ± 0.64	70.21 ± 0.59	0.001*
	CI				0.793 ± 0.039	0.792 ± 0.044	0.839
	HI				1.096 ± 0.017	1.089 ± 0.022	0.09
CTV1_P	D_95_(Gy)	> 61.05Gy	24	25	63.53 ± 0.66	64.41 ± 0.81	< 0.001*
	CI				0.759 ± 0.044	0.728 ± 0.048	0.024
CTV2_P	D_95_(Gy)	> 56.1Gy	24	25	57.11 ± 0.58	57.93 ± 0.66	< 0.001*
	CI				0.856 ± 0.022	0.849 ± 0.021	0.265
GTV_NL_P	D_5_(Gy)				69.17 ± 0.82	68.86 ± 1.10	0.087
	D_95_(Gy)	65.5Gy ~ 67Gy	23	24	66.29 ± 0.40	66.18 ± 0.32	0.169
	CI				0.487 ± 0.100	0.471 ± 0.117	0.961
	HI				1.044 ± 0.017	1.040 ± 0.016	0.587
GTV_NR_P	D_5_(Gy)				69.17 ± 0.59	68.63 ± 0.64	0.003
	D_95_(Gy)	65.5Gy ~ 67Gy	24	25	66.47 ± 0.37	66.25 ± 0.28	0.006
	CI				0.474 ± 0.082	0.484 ± 0.082	0.635
	HI				1.041 ± 0.011	1.036 ± 0.009	0.146
CTV_NL_P	D_95_(Gy)	> 52.8Gy	25	25	54.06 ± 0.51	54.97 ± 0.58	< 0.001*
	CI				0.666 ± 0.036	0.666 ± 0.040	0.884
CTV_NR_P	D_95_(Gy)	> 52.8Gy	25	25	54.18 ± 0.47	54.83 ± 0.59	0.001*
	CI				0.653 ± 0.034	0.653 ± 0.037	0.778
Left parotid	V_30_(%)	< 50%	20	21	45.36 ± 5.06	47.14 ± 4.97	0.091
Right parotid	V_30_(%)	< 50%	20	20	45.75 ± 5.16	47.43 ± 5.13	0.177
Brainstem	D1cc(Gy)	<65Gy	24	23	56.19 ± 6.87	54.95 ± 7.8	0.449
Spinal cord	D1Gy)	<45Gy	23	23	40.23 ± 3.06	40.56 ± 3.59	0.691
Left optic lens	Dmax(Gy)	<8Gy	25	25	5.03 ± 0.87	5.06 ± 0.93	0.861
Right optic lens	Dmax(Gy)	<8Gy	25	25	4.79 ± 0.54	5.02 ± 0.70	0.206
Left optic nerve	Dmax(Gy)	<62Gy	23	23	32.85 ± 19.08	40 ± 20.35	0.677
Right optic nerve	Dmax(Gy)	<62Gy	20	19	33.99 ± 21.58	37.58 ± 21.39	0.473
Pituitary	Dmax(Gy)	<66Gy	17	15	53.55 ± 18.20	55.12 ± 19.17	0.547
Optic chiasm	Dmax(Gy)	<66Gy	21	19	43.52 ± 23.24	44.69 ± 23.8	0.892
MU					685.04 ± 59.63	721.36 ± 63.36	0.051
Duration (min)					9.85 ± 1.13	57.10 ± 6.35	< 0.001*

*ROI* Region of interest, *AP* Automated plan, *MP* Manual plan

*P < 0.0015 indicates statistical significance

All the left and right lens in both the APs and MPs met the dose constraint of Dmax<8Gy. Pituitary appeared the most challenging OAR to manage, as only 17 APs and 15 MPs were able to meet Dmax<66Gy. Notably, the number of the APs was close to that of the MPs in achieving each OAR criterion. The largest different OAR number achieving its criteria between the APs and the MPs was 2 in both the pituitary and optic chiasm..

### Data comparison and analysis

Dose parameters of the PTVs and the OARs using Mann-Whitney U test and Bonferroni correction are also shown in Table 5. PTVs (including GTV_T_P, CTV1_P, CTV2_P, CTV_NL_P, and CTV_NR_P) in the MPs had significantly higher D_95_ than those in the APs (*P* < 0.0015). No significant difference was observed in the D_5_, CI, and HI of PTVs between APs and MPs (*P* > 0.0015). Moreover, dose parameters of all OARs were comparable between APs and MPs (*P* > 0.0015), although all the APs showed lower mean dose parameters (except brainstem D_1cc_) compare to the MPs. The D_1cc_ of brainstem was 56.19 ± 6.87 cGy and 54.95 ± 7.8 cGy in the APs and the MPs, respectively (*P* = 0.449). The MU for the APs was comparable to that for the MPs (685.04 ± 59.63 vs. 721.36 ± 63.36, *P* = 0.051). It was also found that the planning duration for the APs was greatly shorten compared to that for the MPs (9.85 ± 1.13 min vs. 57.10 ± 6.35, P < 0.001).

**Fig. 4:**
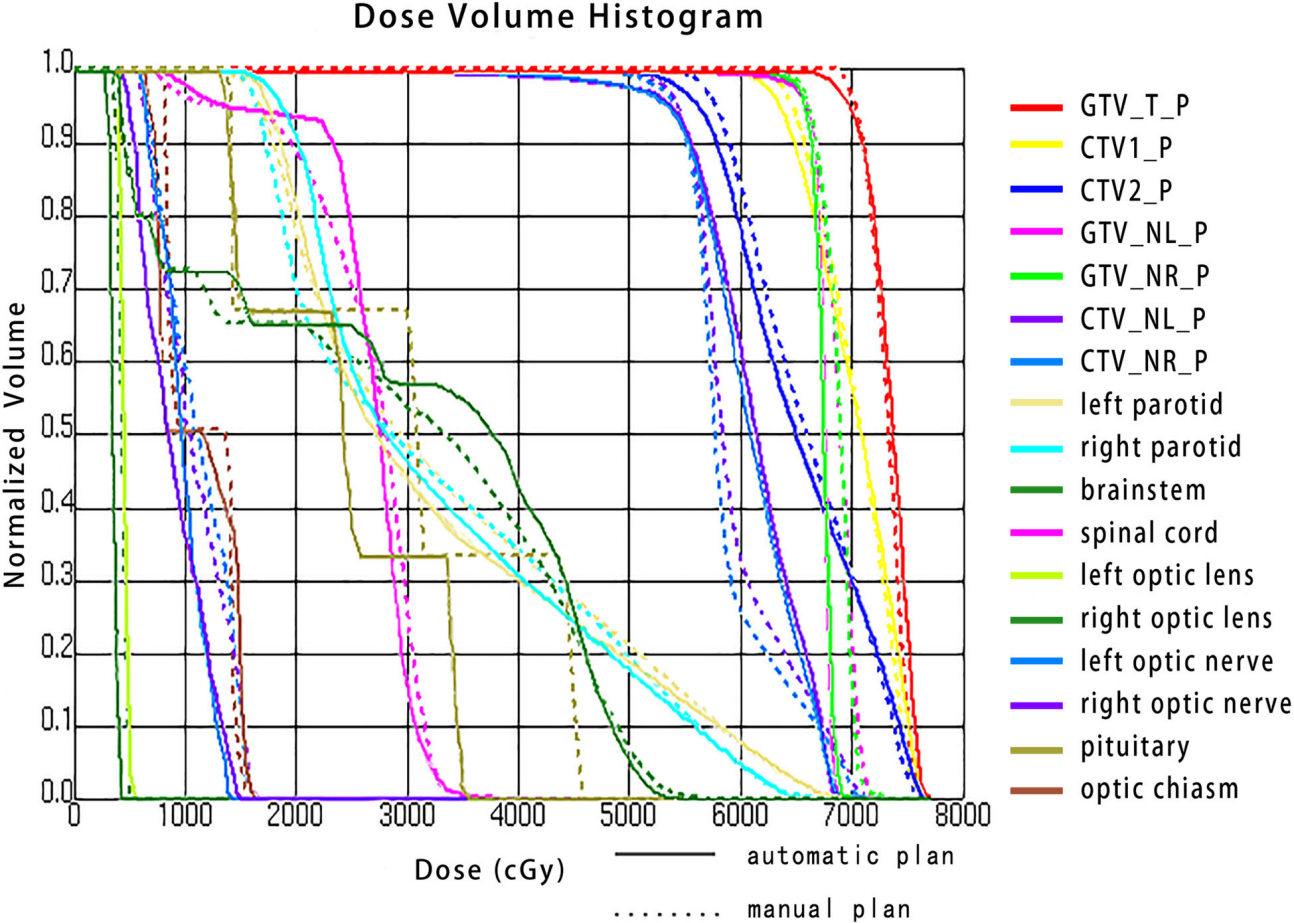
A comparison of dose volume histograms for the automated plan (solid line) and the manual plan (dashed line). As one of the best plans which were both rated grade A, patient (#12) demonstrated acceptable PTV coverage and considerably greater dose sparing to both right optic lens and pituitary

Figure 4 is the DVH for patient (#12), one of the best plans of which its AP (solid line) and MP (dashed line) were both rated grade A. It shows clinically acceptable PTV coverages for both the AP and the MP, and it also shows that the AP considerably increases dose sparing to both right optic lens and pituitary. For patient (#12), the PTV coverage in the AP was approximately equal to that in the MP; relative percentage difference at D_95_ for GTV_T_P, CTV1_P, CTV2_P, GTV_NL_P, GTV_NR_P, CTV_NL_P and CTV_NR_P were − 0.4, − 1.2%, − 2.4, 0.2, 0.2, 1.9 and 1.8%, respectively. Compared to the MP, AP greatly reduced OAR dose for left parotid V_30_, right optic lens Dmax, left optic nerve Dmax, right optic nerve Dmax and pituitary Dmax with relative percentage difference values of − 6.9, − 15.8%, 7.3, 10.2 and 21.2%, respectively.

## Discussion

We developed a feasible knowledge-based IMRT treatment planning technique for locally advanced NPC using a trained 3 layer NN model. The knowledge-based library consisted of a comparatively larger sample size of 115 locally advanced NPC patients [12, 14, 20], and each patient had a high-quality manual IMRT plan. 5-fold cross validation method was also applied in our study. In addition, a wide range of OVH and TVH information which would have a great effect on the resulting dose distribution were selected as the input of the NN model [24]. Patient specific dose objectives predicted by the model were subsequently used for a single-iteration automated planning, which generated high quality, clinically acceptable or superior APs for 21 out of the 25 patients under test. For the 4 patients whose APs were rated C, their MPs were rated C as well (#6, 13, and 18), except for one patient (#25) whose MP was rated B. Further examinations were conducted for these four patients. For the patient (#6), GTV_T_P completely overlapped the left optic nerve. For the patient (#13), GTV_T_P which was given the highest prescription dose overlapped partially with the bilateral parotids, and thus the parotid V_30_ was greatly increased. A large portion of target volume invaded superficial cerebral tissue in the patient (#18), which made it a difficulty to cover the superficial target with the prescription dose. For the above patients, the APs mimicked the manual operation on the choices of optimization priorities. However, the AP for the patient (#25) prioritised pituitary and brainstem and chose to sacrifice the dose coverage on GTV_T_P, in contrast to the MP that well covered the tumor volume. It suggested that our automated technique could not always make expected choices aligning to the oncologist’ preference, particularly for those challenging cases.

Our automated method greatly reduced the planning duration compared to the MPs (9.85 ± 1.13 min vs 57.10 ± 6.35 min). Moreover, it involved no human intervention when the embedded Pinnacle scripts were running. Currently, the dose objectives derived from the NN model on our personal computer had to be manually transferred to the TPS computer, so our knowledge-based automated planning technique was not fully automated in this sense. Nevertheless, the model could be transferred on the TPS to complete the automation workflow in the future. Note that although the training time for each NN model was 27 min, the time to generate a set of objective for one patient took only less than 0.1 s.

Wu B and his group [20] applied k-nearest neighbour method and made a prediction on the best DVH of each single OAR based on its OVH, which might compromise the dose distribution when every OAR reached its best DVH. However, our study took all target volumes and OARs into consideration at the same time, and employed a NN model to derive a patient-specific set of dose objectives.

Our study did not include some OARs such as oral cavity, temporal lobes, and thyroid glands because these OARs could easily achieve their dose constraint by setting dose constraint to the additional rings (R5200, R4500, R3600, and R3100). Our study has not fully addressed the dose inhomogeneity with single iteration optimization. But one study suggested that automatic generation of regions and objectives for hot and cold spots would further improve dose uniformity without manual interference [25]. The study also utilised embedded Pinnacle scripts and provided a solution on achieving better CI and HI for us.

Our study proposed a prospective automated IMRT planning technique for locally advanced NPC. Although our current study has limited the settings of machine parameters such as gantry angles and segment sizes, the same technique can be applied to more complicated IMRT delivery techniques. We anticipate that volumetric modulated arc therapy treatment planning can also take advantage of the described technique to achieve individually tailored optimal radiotherapy plans. In addition, as the volume, position and dose of targets and OARs would change during the treatment course for NPC patients [26–28], the introduction of adaptive radiation therapy (ART) could potentially improve the treatment outcome [29]. Our knowledge-based automated planning approach would be of great value to generate high quality ART plans for NPC patients in an efficient manner.

## Conclusions

A robust and effective knowledge-based IMRT treatment planning technique for locally advanced NPC is developed by use of NN model and HotScripts planning scripts in Pinnacle3 9.2 TPS. This automated technique largely shortened planning time without compromising the plan quality.

## Supplementary information

**Additional file 1.**

## Data Availability

The datasets used and/or analysed during the current study are available from the corresponding author on reasonable request.

## Abbreviations

AP: Automated plan
CI: Conformity index
CT: Computed tomography
CTV: Clinical target volume
DVH: Dose volume histogram
GTV: Gross target volume
HI: Homogeneity index
IMRT: Intensity-modulated radiation therapy
MP: Manual plan
MRI: Magnetic resonance imaging
MU: Monitor unit
NN: Neural network
NPC: Nasopharyngeal carcinoma
OAR: Organ at risk
OVH: Overlap volume histogram
PTV: Planning target volume
ROI: Region of interest
TPS: Treatment planning system
TVH: Target volume histogram
ART: Adaptive radiation therapy
